# Development of a nomogram model to predict malignant vasovagal syncope in Chinese children

**DOI:** 10.3389/fped.2023.1158537

**Published:** 2023-04-03

**Authors:** Rui Sun, Yingying Kang, Mingming Zhang, Hongmao Wang, Lin Shi, Xiaohui Li

**Affiliations:** ^1^ Department of Cardiology, Children's Hospital Capital Institute of Pediatrics, Beijing, China; ^2^ Department of Cardiology, Capital Institute of Pediatrics-Peking University Teaching Hospital, Beijing, China; ^3^ Graduate School of Peking Union Medical College, Capital Institute of Pediatrics, Beijing, China

**Keywords:** malignant vasovagal syncope, head up tilt test, children, risk, nomogram

## Abstract

**Objective:**

Vasovagal syncope (VVS) is the commonest form of syncope, and malignant VVS draws substantial attention due to its life-threatening cardiac asystolic risk. This study aimed to explore the predictive role of a wide panel of clinical indicators for malignant VVS in children, and further to develop a nomogram model.

**Methods:**

This is a retrospective case-control study. VVS is diagnosed based on head-up tilt test (HUTT). STATA software (version 14.0) was used for statistical analysis, and effect sizes are expressed as odds ratio (OR) and 95% confidence interval (CI).

**Results:**

Total 370 children with VVS were analyzed, and of them 16 children had malignant VVS. Sixteen malignant VVS and 64 non-malignant VVS were matched on age and sex by a 1:4 propensity scores matching method. Mean corpuscular hemoglobin (MCH) and standard deviation of average RR intervals milliseconds (SDANN) were significantly and independently associated with malignant VVS after adjusting for confounders, with OR reaching 1.437 (95% CI: 1.044 to 1.979; *P* = 0.026) and 1.035 (95% CI: 1.003 to 1.068; *P* = 0.029), respectively. Calibration and discrimination analyses revealed that the addition of MCH and SDANN can enhance model performance. Then, a nomogram to predict malignant VVS was developed using general characteristics and two above significant factors, and higher values in medical history, number of syncope, MCH and SDANN were associated with a greater risk of malignant VVS.

**Conclusion:**

MCH and SDANN were two promising factors for the development of malignant VVS, and modeling of significant factors in a nomogram can provide strong reference to aid clinical decision-making.

## 1. Introduction

Vasovagal syncope (VVS) is the commonest form of syncope (60%–70%), and its onset peaks initially in childhood and adolescence (1). In routine clinical practice, head-up tilt test (HUTT) is employed to diagnose suspected reflex syncope after preliminary evaluation (2). Based on HUTT technique, there are several types of VVS, and thereof malignant VVS, defined as syncope episodes with ≥3s sinus arrest (3), draws substantial attention, as it can carry the risk of life-threatening cardiac asystolic and seriously impair children's physical and mental health (4, 5).

The incidence rate of sinus arrest during HUTT in pediatric patients with VVS is estimated to be 4.5%–6.5% (6, 7), with the variance possibly attributable to differences in age, race, area, eligibility criteria or test methodology (8). There is evidence that sinus arrest during HUTT can predict spontaneous asystolic syncope in adults ≥ 40 years of age (9). From clinical aspects, early detection of the potential for sinus arrest in children would be helpful for diagnosis and treatment, since it allows for prompt implementation of preventative measures, despite the challenges of performing HUTT at primary hospitals.

Thus far, few studies have attempted to unravel the risk profiles of sinus arrest in children with malignant VVS, whereas the results of these studies are not often reproducible. For example, Dhala and coworkers found that syncope episodes were comparable between subjects with and without sinus arrest, and subjects with sinus arrest tended to be younger (10). Lacroix and coworkers reported no difference in heart rate during supine time and HUTT-positive time between patients with and without sinus arrest (11). In the study by Takase and coworkers, total power was significantly higher in patients with sinus arrest than those without during HUTT (12). Another study by Miranda and coworkers supported the utility of heart rate variability (HRV) in identifying cardioinhibitory syncope (13). Above studies indicated no universal consensus on a certain factor being consistently associated with sinus arrest, likely due to insufficient power to detect significance and simple analysis of limited number of factors in patients with VVS.

To derive a reliable estimate and yield more valuable data for future investigations, we in this clinical study decided to explore the predictive role of a wide panel of clinical indicators for malignant VVS in children, and further to develop a nomogram model to facilitate application in routine clinical practice.

## 2. Methods

### 2.1. Study design and ethical aspects

This is a retrospective case-control study. Ethical approval was obtained from the ethics committee of the Capital Institute of Pediatrics (Approval No. SHERLLM2021025).

### 2.2. Study participants

All study participants diagnosed with VVS were retrospectively enrolled from the Department of Cardiology, Children's Hospital Capital Institute of Pediatrics during the period from July 2017 to August 2022.

According to medical records, patients with a clinical diagnosis of VVS were abstracted. In patients with VVS, malignant VVS is defined as syncope episodes with ≥ 3s sinus arrest during HUTT.

### 2.3. Eligibility criteria

Patients must satisfy the following four criteria: (1) children and adolescents under 18 years of age; (2) with a positive history of syncope; (3) with positive HUTT results.

In addition, patients were excluded if they had one or more of the following: (1) cardiopulmonary diseases, such as cardiomyopathy, arrhythmia and congenital heart defects or hypertension; (2) neuropsychological disorder, such as depression and anxiety, epilepsy and so on; (3) genetic metabolic diseases, such as hyperthyroidism, hereditary channelopathies (e.g., brugada syndrome, long QT syndrome, et al.) and so on;(4) any other diagnosis during HUTT, such as orthostatic hypotension, orthostatic hypertension, or the postural orthostatic tachycardia syndrome.

### 2.4. Head-up tilt test

The HUTT test was performed in a serene setting with low lighting. Cuff blood pressure (BP) and heart rate (HR) was continuously monitored utilizing an electric tilting device (SHUT-100A, STANDARD, Beijing, China). After an initial observation of patient in the supine position for 10 min. The patients were on a 45-min tilt phase without medication at a 60° tilt. If syncope does not occur, administer sublingual nitroglycerin (4–6 μg/kg, maximum ≤ 0.3 mg) for 20 min. If syncope occurs during the test, tilt table is quickly lowered to the prone position. In addition to four key factors, a positive response to HUTT involves syncope or presyncope, including (1) a drop in blood pressure (systolic blood pressure ≤ 80 mmHg, diastolic blood pressure ≤ 50 mmHg, or a mean decrease in blood pressure ≥ 25%); (2) sinus arrest with a junctional escape rhythm; (3) atrioventricular block ≥ II° or cardiac arrest for 3 s; (4) the heart rate of children aged 4–6 is decreased < 75 bpm, and the heart rate of children aged 7–8 is decreased < 65 bpm(14).

### 2.5. Data collection

Baseline characteristics from assessable children admitted for the first time to our hospital were collected, including age, sex, body mass index (BMI), medical history, family history of syncope, number of syncope, from medical records. Medical history is defined as the first symptoms of syncope or presyncope presented in our hospital.

In addition, blood tests, cardiac enzyme markers, 24-h urine output, 24-h Holter and echocardiography were completed and collected before HUTT. The supine BP/HR was recorded when the subject was in that position, and the tilt BP/HR was obtained during the first minute after tilt, when the subject was stable. All examinations are conducted by the auxiliary departments of our hospital.

### 2.6. Statistical analysis

Study children were grouped into malignant VVS and non-malignant VVS patients. To balance between-group bias in sample sizes, a 1:4 propensity score matching (PSM) analysis was conducted by equating groups based on age and sex. Continuous data are expressed as median (interquartile range) and compared using the Mann-Whitney test in case of deviation from normal distributions and as mean (standard deviation) and using the t-test otherwise. Number (percent) is used to express categorical data and the *χ*^2^ test or Fisher test was used for between-group comparison.

Univariate Logistic regression analyses were used to identify single factors in significant association with malignant VVS, and then multivariable Logistic regression analyses were used to check the independent association of these significant factors after adjusting for age, sex, BMI, number of syncope, family history of syncope and medical history. Effect sizes are expressed as odds ratio (OR) and 95% confidence interval (95% CI).

Predication performance gained by adding significant factors identified aforementioned to the basic model (including age, sex, medical history, number of syncope and family history of syncope) was appraised from both calibration and discrimination aspects. Correlation analysis was done to quantify the magnitude of collinearity across factors under study. Regarding corrections, Akaike Information Criterion (AIC) (15), Bayesian Information Criterion (BIC) (16) and likelihood ratio tests were used to assess the global fit of predicted probability to the reflectance of the actual observed risk and the revised risk model, by adding significant factors identified. The lower values of AIC and BIC indicate a better model. From the discrimination aspects, net reclassification improvement (NRI), integrated discrimination improvement (IDI), change in area under the curve (*Δ*AUC) and decision curve analysis (DCA) were used to evaluate the clinical benefits and utility of the full model relative to the basic model (17–19). DCA can calculate the net benefit of models across various risk thresholds by taking account of weighted risks and benefits. The net benefit increases with distance from both the horizontal line with no malignant VVS and the solid curve line under all malignant VVS.

Finally, a nomogram model in predicting malignant VVS risk was developed, and the predictive accuracy of this model was quantified by concordance index (C-index), defined as the area under the receiver operating characteristics curve. The nomogram and accuracy assessment were implemented by the regression modeling strategies (RMS) package (available at the website https://cran.r-project.org/web/packages/rms/index.html) in R programming environment (version 4.2.1).

All statistical analyses were conducted using the Stata software (version 14.0, Stata Corp, TX, USA) unless otherwise indicated. A two-sided *P* value less than 0.05 was considered statistically significant.

## 3. Results

### 3.1. Characteristics of study patients

**Figure 1:**
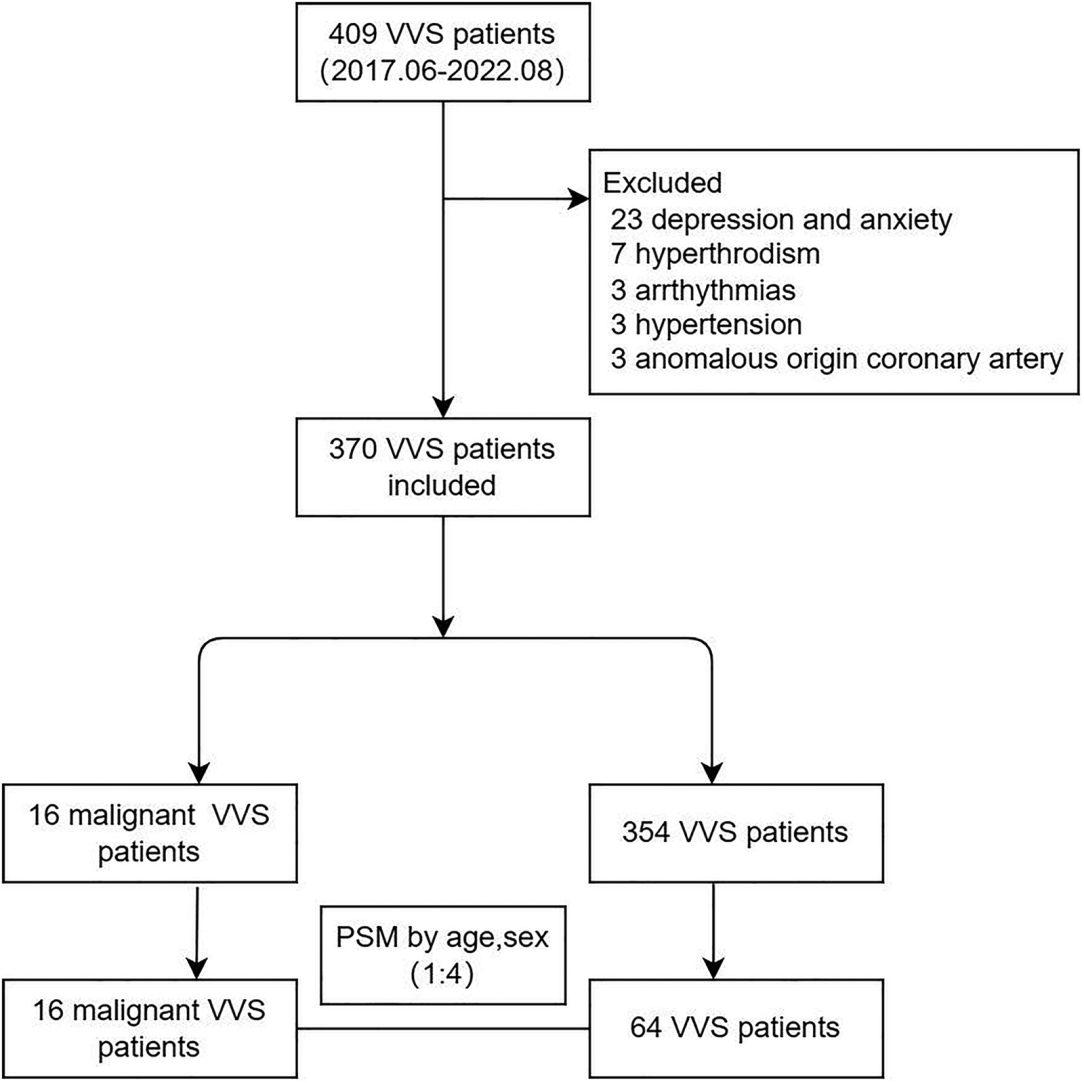
Flow chart of enrolled participants. Abbreviations: PSM, propensity score matching.

Among 409 children diagnosed with VVS in this study, 37 were eliminated due to depression and anxiety (*n* = 23), hyperthyroidism (*n* = 7), arrhythmias (*n* = 3), hypertension (*n* = 3) and anomalous origin coronary artery (*n* = 3). Ultimately, total 370 children with VVS were analyzed, and of them 16 children had malignant VVS. (Figure 1) Children with malignant VVS had a median age of 10 [interquartile range (IQR): 8.98 to 12.34] years, significantly lower than that of 354 children free of malignant VVS (*P* < 0.05). The baseline characteristics of children with and without malignant VVS are compared in Supplementary Table S1.

Considering the wide difference in sample size between children with and without malignant VVS, 64 children with non-malignant VVS were extracted by means of PSM technique to match 16 children with malignant VVS on age and sex. After 1:4 PSM, the comparison of baseline characteristics between 16 children with malignant VVS and 64 children with non-malignant VVS is displayed in Table 1. Medical history and the number of syncope in history differed significantly different between the two groups (both *P* < 0.05) (Table 1).

**Table 1:** Baseline characteristics of study children after propensity score matching.

Characteristics	Non-malignant VVS (*n* = 64)	Malignant VVS (*n* = 16)	*P*
Age (years)	10.64 [8.39, 12.24]	10.45 [8.70, 12.25]	0.933
Sex (%)			1.000
Female	41 (64,1)	10 (62.5)	
Male	23 (35.9)	6 (37.5)	
Body-mass index (kg/m^2^)	17.11 [15.52, 18.87]	16.09 [15.70, 17.92]	0.661
Medical history (months)	2.00 [0.48, 15.00]	30.00 [11.00, 48.00]	0.001
Number of syncope	1.00 [0.00, 3.00]	4.00 [1.75, 5.00]	0.001
Family history of syncope (%)			0.685
Without	56 (87.5)	13 (81.2)	
With	8 (12.5)	3 (18.8)	
Hemoglobin (g/L)	129.00 [123.00, 136.00]	132.00 [127.50, 140.25]	0.206
MCV (fL)	83.80 [81.20, 86.90]	84.30 [82.65, 85.40]	0.714
MCH (pg)	28.60 [27.85, 29.60]	29.50 [28.32, 30.35]	0.117
MCHC (g/L)	343.00 [337.00, 346.00]	345.00 [340.75, 350.25]	0.1
Creatine Kinase (U/L)	75.00 [62.00, 91.50]	73.00 [61.75, 77.50]	0.542
Creatine Kinase-MB (ng/ml)	0.57 [0.25, 0.95]	0.81 [0.40, 1.10]	0.235
Urine output (ml/24 h)	1322.50 [996.00, 1581.75]	1292.50 [779.25, 1446.50]	0.351
Total-Na (mmol/24 h)	114.16 [79.40, 153.19]	139.25 [82.68, 151.86]	0.572
Total-K (mmol/24 h)	28.98 [22.76, 36.33]	29.91 [26.95, 35.14]	0.647
Urine specific gravity	1.01[1.01, 1.02]	1.02[1.02, 1.03]	0.040
LVEF (%)	71.00 [67.75, 73.00]	70.00 [68.00, 72.00]	0.507
LVFS (%)	40.00 [36.75, 42.00]	39.00 [37.00, 41.00]	0.524
**24-H Holter**			
Average HR (bpm)	83.00 [77.50, 88.50]	78.00 [71.00, 85.00]	0.168
SDNN (ms)	147.50 [122.50, 162.25]	141.00 [136.00, 161.50]	0.457
SDANN (ms)	123.00 [105.00, 137.00]	124.00 [120.00, 143.00]	0.245
SDNN index (ms)	75.00 [61.50, 89.00]	73.00 [63.00, 93.00]	0.824
pNN50 (%)	23.00 [17.50, 34.00]	25.00 [22.50, 34.00]	0.492
DC	7.00 [6.50, 7.75]	7.00 [6.40, 8.05]	0.797
TP	4145.70 [2814.00, 5518.50]	4458.00 [3599.50, 7109.35]	0.151
LF/HF	1.44 [1.09, 1.89]	1.66 [1.23, 2.04]	0.321
**Head-up Tilt Test**			
Supine SBP (mm Hg)	110.00 [101.75, 116.50]	109.00 [100.00, 113.50]	0.460
Supine DBP (mm Hg)	63.00 [60.00, 68.00]	65.00 [58.50, 68.00]	0.846
Supine HR (bpm)	75.50 [71.00, 83.25]	74.00 [64.25, 81.00]	0.185
Tilt SBP (mm Hg)	110.00 [102.75, 120.25]	109.00 [104.00, 116.50]	0.586
Tilt DBP (mm Hg)	65.50 [61.00, 72.00]	67.00 [61.50, 74.00]	0.759
Tilt HR (mm Hg)	94.00 [80.00, 103.25]	88.50 [75.75, 99.25]	0.234
Nitroglycerin (%)			0.386
Without	22 (34.4)	8 (50)	
With	42 (65.6)	8 (50)	

VVS, vasovagal syncope; SBP, systolic blood pressure; DBP, diastolic blood pressure; Tilt: mean the tilt immediately; HR, heart rate; MCV, mean corpuscular volume; MCH, mean corpuscular hemoglobin; MCHC, mean corpuscular hemoglobin concentration; LVEF, left ventricular ejection fraction; LVFS, left ventricular fraction shortening; SDNN, standard deviation of RR intervals in milliseconds; SDANN, standard deviation of the average RR intervals in milliseconds; SDNN index, mean score of the standard deviations of all RR intervals in 5-min segments in milliseconds; pNN50, proportion of pairs of successive RR intervals differing by more than 50 ms divided by the total number of RR intervals (percentage); DC, deceleration capacity; TP, total power is the frequency components in heart rate variability; LF/HF, ratio between the low- and high frequency component.

### 3.2. Identification of predictors for malignant VVS

Two factors—mean corpuscular hemoglobin (MCH) and standard deviation of average RR intervals milliseconds (SDANN), were found to be significantly and independently associated with the risk of malignant VVS after adjusting age, sex, BMI, medical history, number of syncope and family history of syncope factors, with OR reaching 1.437 (95% CI: 1.044 to 1.979; *P* = 0.026) and 1.035 (95% CI: 1.003 to 1.068; *P* = 0.029), respectively (Table 2).

**Table 2:** Identification of potential factors for malignant VVS before and after adjustment.

Variables	Unadjusted model			Adjusted model		
	OR	95% CI	*P*	OR	95% CI	*P*
Age	0.995	0.782 to 1.267	0.970	NA	NA	NA
Male	1.070	0.344 to 3.323	0.907	NA	NA	NA
BMI	0.983	0.810 to 1.231	0.990	NA	NA	NA
**Medical history**	**1**.**064**	**1.028 to 1.101**	**<0**.**001**	NA	NA	NA
**Number of syncope**	**1**.**656**	**1.221 to 2.246**	**0**.**001**	NA	NA	NA
Family history of syncope	1.625	0.376 to 6.940	0.519	NA	NA	NA
Hemoglobin	1.025	0.969 to 1.084	0.388	1.041	0.963 to 1.125	0.312
MCV	1.024	0.867 to 1.208	0.781	1.169	0.924 to 1.479	0.193
**MCH**	1.197	0.916 to 1.563	0.188	**1**.**437**	**1.044 to 1.979**	**0**.**026**
MCHC	1.065	0.993 to 1.142	0.078	1.055	0.966 to 1.152	0.232
Creatine Kinase	0.997	0.977 to 1.017	0.739	0.993	0.964 to 1.021	0.609
Creatine Kinase-MB	1.715	0.553 to 5.322	0.351	1.292	0.498 to 3.350	0.598
LVEF	0.960	0.841 to 1.096	0.544	0.961	0.810 to 1.139	0.645
LVFS	0.953	0.812 to 1.118	0.556	0.958	0.781 to 1.175	0.680
Urine output of 24 h	0.999	0.998 to 1	0.204	0.999	0.998 to 1	0.189
Total-Na	1.003	0.992 to 1.014	0.615	0.998	0.982 to 1.014	0.769
Total-K	0.999	0.946 to 1.054	0.963	1.025	0.963 to 1.092	0.435
Urine specific gravity	1.092	1.003 to 1.190	0.043	1.085	0.975 to 1.209	0.135
Average HR (bpm)	0.960	0.906 to 1.017	0.168	0.943	0.865 to 1.028	0.183
SDNN	0.999	0.993 to 1.005	0.797	1.001	0.995 to 1.006	0.792
**SDANN**	1.014	0.993 to 1.036	0.187	**1**.**035**	**1.003 to 1.068**	**0**.**029**
SDNN index	0.997	0.984 to 1.010	0.661	0.999	0.987 to 1.012	0.915
pNN50	1.010	0.964 to 1.072	0.547	1.016	0.964 to 1.072	0.547
DC	1.049	0.657 to 1.676	0.841	0.573	0.274 to 1.200	0.140
TP	1	1 to 1	0.494	1	1 to 1	0.311
LF/HF	1.29	0.578 to 2.879	0.534	2.056	0.690 to 6.127	0.196
Supine SBP (mm Hg)	0.962	0.912 to 1.015	1.015	0.977	0.904 to 1.055	0.551
Supine DBP (mm Hg)	0.990	0.909 to 1.077	0.809	0.991	0.898 to 1.095	0.866
Supine HR (bpm)	0.962	0.914 to 1.012	0.130	0.982	0.921 to 1.038	0.586
Tilt SBP (mm Hg)	0.987	0.941 to 1.035	0.587	0.432	0.918 to 1.037	0.432
Tilt DBP (mm Hg)	1.009	0.943 to 1.079	0.801	1.008	0.936 to 1.086	0.839
Tilt HR (bpm)	0.977	0.943 to 1.013	0.202	0.978	0.936 to 1.022	0.326
Nitroglycerin	0.599	0.192 to 1.868	0.377	0.616	0.154 to 2.473	0.495

SBP, systolic blood pressure; DBP, diastolic blood pressure; HR, heart rate; MCV, mean corpuscular colume; MCH, mean corpuscular hemoglobin; MCHC, mean corpuscular hemoglobin concentration; LVEF, left ventricular ejection fraction; LVFS, left ventricular fraction shortening; SDNN, standard deviation of RR intervals in milliseconds; SDANN, standard deviation of the average RR intervals milliseconds; SDNN index, mean score of the standard deviations of all RR intervals in 5-min segments in milliseconds; pNN50, Proportion of pairs of successive RR intervals differing by more than 50 ms divided by the total number of RR intervals (percentage); DC, deceleration capacity; TP, total *p*ower is the frequency components in heart rate variability; LF/HF, ratio between the low- and high frequency component; Adjusted factors include age, sex, BMI, medical history, number of syncope, family history.

### 3.3. Calibration and discrimination assessment

Two models were built to assess prediction performance of two significant factors identified: the basic model and the full model. The basic model includes age, sex, medical history, number of syncope and family history of syncope, and the full model additionally includes MCH and SDANN.

As shown in Table 3, according to stepwise regression results (Table 2, Supplementary Table S2), the full model had minimal AIC and BIC. No collinearity was noticed among the variables in full model (Supplementary Figure S1). The predictive performance of the two models was significantly different by the likelihood ratio test (*P* = 0.013). NRI and IDI statistics showed significant improvement after adding MCH and SDANN to the basic model.

**Table 3:** Predictive accuracy in models with and without two significant factors when predicting malignant VVS.

Statistics	Basic model	Full model
***Calibration***		
AIC	70.532	62.420
BIC	82.442	78.453
LR test (*χ*^2^)	8.6	
LR test (*P* value)	0.013	
***Discrimination***		
NRI	0.067	
IDI	0.002	

Basic model include age, sex, medical history, number of syncope; Full model include age, sex, medical history, number of syncope, family history of syncope; MCH, mean corpuscular hemoglobin; SDANN, standard deviation of the average NN intervals); VVS, vasovagal syncope; AIC, akaike information criterion; BIC, bayesian information criteria; LR test, likelihood ratio test; NRI, net reclassification improvement; IDI, integrated discrimination improvement.

**Figure 2:**
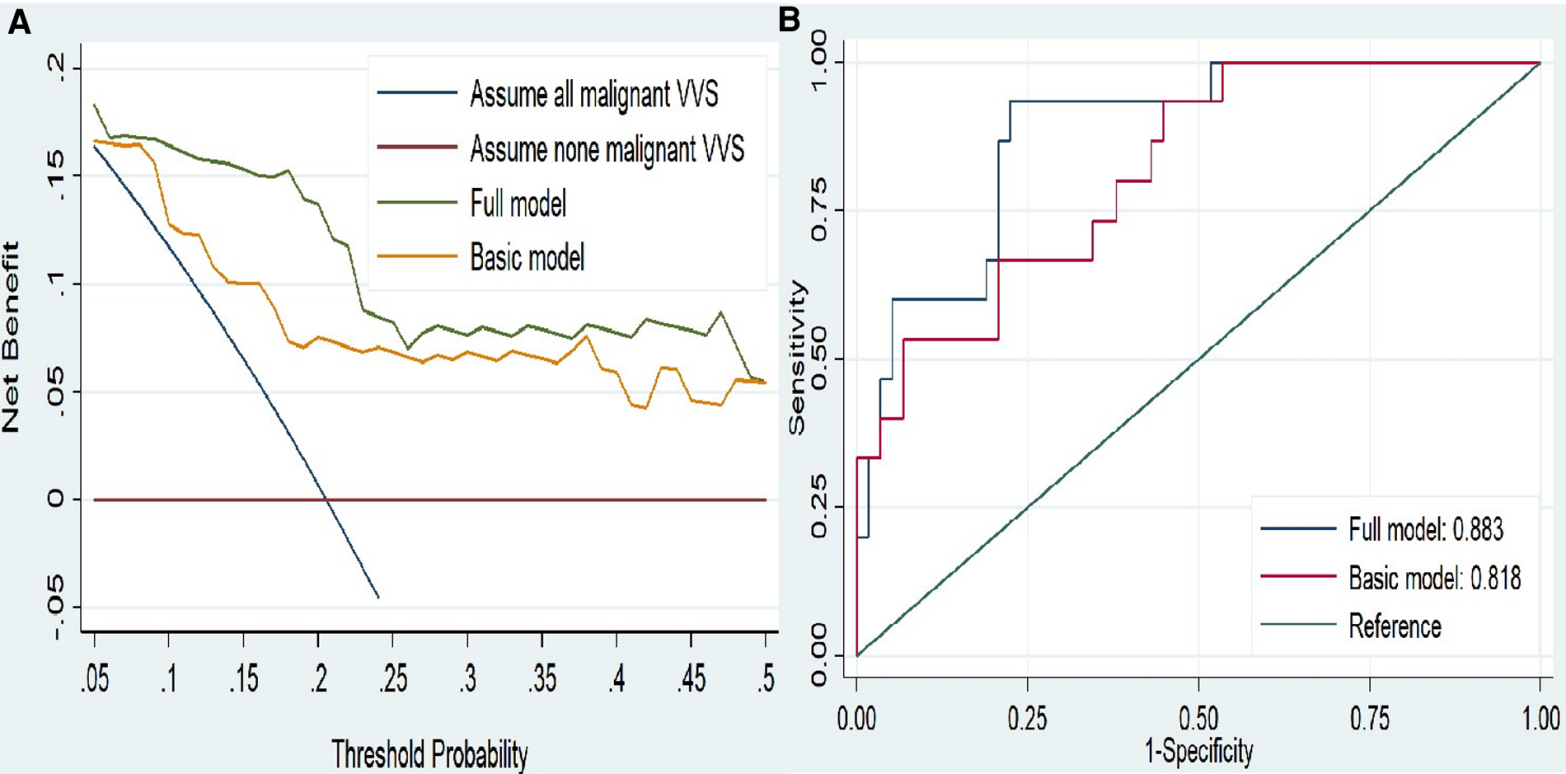
(**A**) decision curves analysis for malignant vasovagal syncope. (**B**)ROC curves for the two model. The basic model included age, sex, body mass index, medical history, number of syncope and family history. The full model included indicators in basic model and add MCH and SDANN. Abbreviations: MCH, mean corpuscular hemoglobin; SDANN, standard deviation of the average NN intervals.

Furthermore, DCA indicated the net benefits gained by adding the two significant factors (MCH and SDANN) to the basic model (Figure 2A). Addition of the two significant factors increased the AUC from 0.818 to 0.883, reflecting 6.5 percentage improvement in AUC (Figure 2B).

### 3.4. Nomogram development

**Figure 3:**
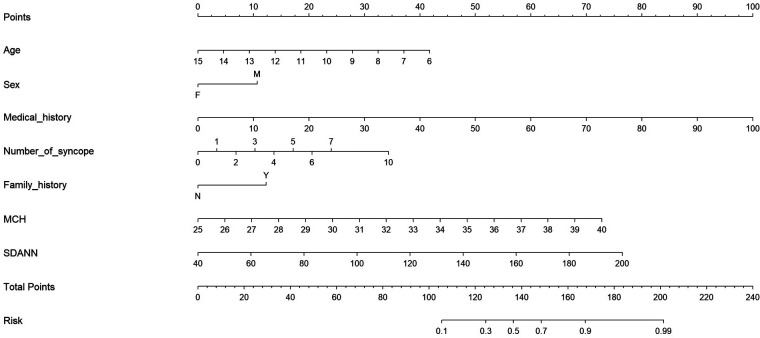
Prediction nomogram of convenient and significant factors for malignant VVS. Abbreviations: Medical history, the medical history of VVS (counts in months); F, female; M, male; MCH, mean corpuscular hemoglobin; SDANN, standard deviation of the average RR intervals in milliseconds.

A nomogram to predict malignant VVS was developed using the general characteristics and two significant factors (Figure 3). In the nomogram, higher values in medical history, number of syncope, MCH and SDANN were associated with a greater risk of malignant VVS. For example, assuming a child with a medical history at 50 months (50 points), number of syncope at 5 (20 points), family history of syncope (12 points), 10 years old (22 points), female (0 points), MCH at 35.5 pg (50 points) and SDANN at 125 ms (40 points), the probability of malignant VVS was estimated to be 97%.

The nomogram model demonstrated a good accuracy for predicting malignant VVS, with a C-index of 0.883 and calibration curves of good consistency (Supplementary Figure S2).

## 4. Discussion

The aim of this study was to explore the predictive role of a wide panel of clinical indicators for malignant VVS in children in clinical settings. Our key findings are that two clinical predictors, MCH and SDANN, were significantly and independently associated with the risk of malignant VVS in children. Moreover, integration of convenient characteristics and two significant factors in a nomogram can decently predict malignant VVS. To our knowledge, this study has for the first time developed a prediction nomogram model based on clinical data for malignant VVS in the literature.

Prior studies on malignant VVS suggested some factors can impact its occurrence, such as age and sex (20, 21), which were considered in the present study. In addition, there is evidence that with aging, cardioinhibitory decreased and asystolic pauses occurred less frequently (20), consistent with the results of another study in adult patients (22, 23). By contrast, our findings in children supported these observations, that is, older children were less likely to experience malignant VVS, which could be explained by the hypothesis that autonomic function development is not yet complete in children with malignant VVS at a young age. Furthermore, malignant VVS was found to be associated with a relatively low heart rate at baseline (23), which was not observed in the present study, possibly due to the differences in the characteristics of study participants. As an extension of prior studies, we found that medical history and the number of previous syncope were linked to malignant VVS, which led us to speculate that the likelihood of malignant VVS occurrence increased with the length of disease history.

In this study, it is worth noting that two factors, MCH and SDANN, were in significant and independent association with malignant VVS. On one hand, MCH is a red blood cell index and it is a primary indicator of red blood cell development and hemoglobin content. As indicated by our findings, high levels of MCH were found to be associated with high risk of malignant VVS. From genetic point of view, RBC indices correlated with the expression of many genes such as RPN1, ELL2, MIDN, HBB, HBA1, PIEZO1 and G6PD (24), while these genes are not among the list of candidate genes for VVS (25, 26). We agree that candidate gene approaches focusing on these RBC-correlated genes in susceptibility to VVS or malignant VVS are encouraging. Additionally, increased MCH may be caused by related hypovolemia.

On the other hand, SDANN, one time-domain indicator of HRV, reflects sympathetic nerve activity (27). Previous studies reported the diagnostic value of HRV for VVS (28). In fact, high SDANN in 24-h Holter before sinus arrest occurred can indicate that children with malignant VVS have higher activity of sympathetic nerve, which may involve the pathogenesis of malignant VVS. Our study also found that a decrease in 24 h urine output and an increase in urine specific gravity were risk factors for malignant VVS (OR: 0.993 and 1.085, respectively), albeit no hints of statistical significance. Nonetheless, this finding suggested that hypovolemia was a risk factor for malignant VVS in the case of normal renal function in children. Altogether, these findings indicate that blood volume, HRV and genetic factors are related to malignant VVS. Considering the fact that the etiology of malignant VVS is complex and the relative risk attributable to a single factor is small.

Besides the obvious strengths of this study, including the successful internal validation and good calibration/discrimination scores, some potential limitations should be recognized. First, this study was conducted at a single center involving a small sample size, and further larger multicenter studies will be required to validate our findings. Second, the presyncope symptoms and accompanying symptoms during syncope were unavailable for us, and so we cannot distinguish malignant VVS symptomatically. However, considering the subjective nature of symptom presentation, we chose a more objective laboratory basis. Third, this study is retrospective in design, and a recall bias cannot be fully excluded. Last but not the least, all clinical indicators were only measured once at baseline, and may not reflect long-term circulating concentrations.

## 5. Conclusions

To sum up, our findings indicated that MCH and SDANN were two promising factors for the development of malignant VVS, and modeling of significant factors in a nomogram can provide strong reference to aid clinical decision-making. In the long run, past and current findings, annexed with advanced analytical strategies such as artificial intelligence techniques, will provide novel insights about the risk profiles of malignant VVS.

## Data Availability

The original contributions presented in the study are included in the article/**Supplementary Material**, further inquiries can be directed to the corresponding author/s.
