# Supplementary material for: Development of a nomogram model to predict malignant vasovagal syncope in Chinese children
Source: Front Pediatr. 2023 Apr 3;11:1158537. doi: 10.3389/fped.2023.1158537 (PMC10109463; doi:10.3389/fped.2023.1158537)
Supplement: Supplementary file 1 [file Datasheet1.docx]

Supplementary Material

1. Supplementary Figures and Tables

| **Table 1S.** Baseline characteristics of study children before propensity score matching. | | | |
| --- | --- | --- | --- |
| **Characteristics** | **VVS (n=354)** | **Malignant VVS (n=16)** | ***P*** |
| Age (years) | 12.12 [9.68, 13.53] | 10.45 [8.70, 12.25] | 0.039 |
| Sex (%) |  |  | 0.934 |
| Female | 206 (58.2) | 10 (62.5) |  |
| Male | 148 (41.8) | 6 (37.5) |  |
| Body-mass index (kg/m^2^) | 17.94 [15.80, 20.06] | 16.09 [15.70, 17.92] | 0.13 |
| Medical history (months) | 4.00 [1.00, 15.75] | 30.00 [11.00, 48.00] | 0.001 |
| Number of syncope | 1.00 [0.00, 2.00] | 4.00 [1.75, 5.00] | <0.001 |
| Family history of syncope (%) |  |  | 0.408 |
| Without | 315 (89) | 13 (81.2) |  |
| With | 39 (11) | 3 (18.8) |  |
| Hemoglobin (g/L) | 130.00 [124.00, 138.00] | 132.00 [127.50, 140.25] | 0.414 |
| MCV (fL) | 84.40 [81.90, 87.00] | 84.30 [82.65, 85.40] | 0.797 |
| MCH (pg) | 28.70 [27.80, 29.70] | 29.50 [28.32, 30.35] | 0.097 |
| MCHC (g/L) | 339.00 [332.00, 345.00] | 345.00 [340.75, 350.25] | 0.011 |
| Creatine Kinase (U/L) | 73.00 [58.75, 95.00] | 73.00 [61.75, 77.50] | 0.627 |
| Creatine Kinase-MB (ng/ml) | 0.50 [0.20, 0.83] | 0.81 [0.40, 1.10] | 0.029 |
| Urine output (ml/24h) | 1324.00 [900.00, 1766.00] | 1292.50 [779.25, 1446.50] | 0.243 |
| Total-Na (mmol/24h) | 119.88 [86.07, 171.97] | 139.25 [82.68, 151.86] | 0.993 |
| Total-K (mmol/24h) | 30.79 [23.59, 39.45] | 29.91 [26.95, 35.14] | 0.784 |
| Urine specific gravity | 1.02 [1.01, 1.02] | 1.02 [1.02, 1.02] | 0.290 |
| LVEF (%) | 70.00 [67.00, 73.00] | 70.00 [68.00, 72.00] | 0.907 |
| LVFS (%) | 39.00 [36.00, 42.00] | 39.00 [37.00, 41.00] | 0.978 |
| **24-Hour Holter** |  |  |  |
| Average HR | 81.00 [75.00, 87.00] | 78.00 [71.00, 85.00] | 0.441 |
| SDNN (ms) | 151.00 [127.00, 175.00] | 141.00 [136.00, 161.50] | 0.851 |
| SDANN (ms) | 127.00 [108.75, 149.25] | 124.00 [120.00, 143.00] | 0.656 |
| SDNN index (ms) | 72.00 [60.00, 88.00] | 73.00 [63.00, 93.00] | 0.523 |
| pNN50 (%) | 22.00 [15.00, 32.00] | 25.00 [22.50, 34.00] | 0.235 |
| DC | 7.12 [6.30, 8.00] | 7.00 [6.40, 8.05] | 0.836 |
| TP | 4099.70 [3009.95, 5751.05] | 4458.00 [3599.50, 7109.35] | 0.153 |
| LF/HF | 1.52 [1.19, 2.02] | 1.66 [1.23, 2.04] | 0.642 |
| **Head-up Tilt Test** |  |  |  |
| Supine SBP (mm Hg) | 111.00 [103.00, 118.00] | 109.00 [100.00, 113.50] | 0.236 |
| Supine DBP (mm Hg) | 65.00 [60.00, 69.00] | 65.00 [58.50, 68.00] | 0.555 |
| Supine HR (bpm) | 76.00 [69.00, 84.00] | 74.00 [64.25, 81.00] | 0.167 |
| Tilt SBP (mm Hg) | 111.00 [103.00, 121.00] | 109.00 [104.00, 116.50] | 0.483 |
| Tilt DBP (mm Hg) | 68.00 [63.00, 74.00] | 67.00 [61.50, 74.00] | 0.813 |
| Tilt HR (bpm) | 93.00 [84.00, 104.00] | 88.50 [75.75, 99.25] | 0.122 |
| Positive reaction time (min) | 35.00 [20.00, 35.00] | 33.00 [14.00, 35.00] | 0.205 |
| Nitroglycerin (%) |  |  | 0.408 |
| Without | 131 (37) | 8 (50) |  |
| With | 223 (63) | 8 (50) |  |

Abbreviations: SBP, systolic blood pressure; DBP, diastolic blood pressure; HR, heart rate; Tilt: mean tilt immediately; MCV, Mean Corpuscular Volume; MCH, Mean Corpuscular Hemoglobin; MCHC, mean corpuscular hemoglobin concentration; LVEF, left ventricular ejection fraction; LVFS, left ventricular fraction shortening; SDNN, standard deviation of RR intervals in milliseconds; SDANN, Standard deviation of the average RR intervals milliseconds; SDNN index, Mean score of the standard deviations of all RR intervals in 5-min segments in milliseconds; pNN50, Proportion of pairs of successive RR intervals differing by more than 50ms divided by the total number of RR intervals (percentage); DC, Deceleration Capacity; TP, Total Power is the frequency components in heart rate variability; LF/HF, Ratio between the low and high frequency component.

**Table 2S.** Identification of potential factors for malignant VVS after adjustment of age, sex, and BMI.

| **Variables** | **OR** | **95% CI** | ***P*** |
| --- | --- | --- | --- |
| Medical history | 1.066 | 1.029 to 1.105 | 0.000 |
| Number of syncope | 1.693 | 1.237 to 2.318 | 0.001 |
| Family history of syncope | 1.600 | 0.363 to 7.049 | 0.534 |
| Hemoglobin | 1.026 | 0.966 to 1.089 | 0.399 |
| MCV | 1.045 | 0.855 to 1.276 | 0.670 |
| MCH | 1.246 | 0.927 to 1.673 | 0.145 |
| MCHC | 1.066 | 0.992 to 1.147 | 0.082 |
| Creatine Kinase | 0.995 | 0.974 to 1.017 | 0.680 |
| Creatine Kinase-MB | 1.696 | 0.626 to 4.593 | 0.298 |
| LVEF | 0.959 | 0.840 to 1.095 | 0.535 |
| LVFS | 0.953 | 0.812 to 1.118 | 0.553 |
| Urine output of 24h | 0.999 | 0.998 to 1.000 | 0.167 |
| Urine specific gravity | 1.096 | 1.004 to 1.198 | 0.041 |
| Average HR (bpm) | 0.930 | 0.859 to 1.008 | 0.077 |
| SDNN | 0.999 | 0.993 to 1.005 | 0.791 |
| SDANN | 1.019 | 0.995 to 1.044 | 0.125 |
| SDNN index | 0.997 | 0.984 to 1.010 | 0.647 |
| pNN50 | 1.011 | 0.965 to 1.059 | 0.648 |
| DC | 1.049 | 0.653 to 1.684 | 0.844 |
| TP | 1.000 | 1.000 to 1.000 | 0.409 |
| LF/HF | 1.355 | 0.568 to 3.228 | 0.493 |
| Supine SBP (mm Hg) | 0.959 | 0.906 to 1.014 | 0.144 |
| Supine DBP (mm Hg) | 0.989 | 0.908 to 1.076 | 0.793 |
| Supine HR (bpm) | 0.955 | 0.901 to 1.011 | 0.115 |
| Tilt SBP (mm Hg) | 0.985 | 0.937 to 1.035 | 0.546 |
| Tilt DBP (mm Hg) | 1.007 | 0.940 to 1.079 | 0.833 |
| Tilt HR (bpm) | 0.976 | 0.940 to 1.012 | 0.191 |
| Nitroglycerin | 0.580 | 0.183 to 1.838 | 0.355 |

Abbreviations: SBP, systolic blood pressure; DBP, diastolic blood pressure; HR, heart rate; MCV, Mean Corpuscular Volume; MCH, Mean Corpuscular Hemoglobin; MCHC, mean corpuscular hemoglobin concentration; LVEF, left ventricular ejection fraction; LVFS, left ventricular fraction shortening; SDNN, standard deviation of RR intervals in milliseconds; SDANN, Standard deviation of the average RR intervals milliseconds; SDNN index, Mean score of the standard deviations of all RR intervals in 5-min segments in milliseconds; pNN50, Proportion of pairs of successive RR intervals differing by more than 50ms divided by the total number of RR intervals (percentage); DC, Deceleration Capacity; TP, Total Power is the frequency components in heart rate variability ; LF/HF, Ratio between the low and high frequency component.


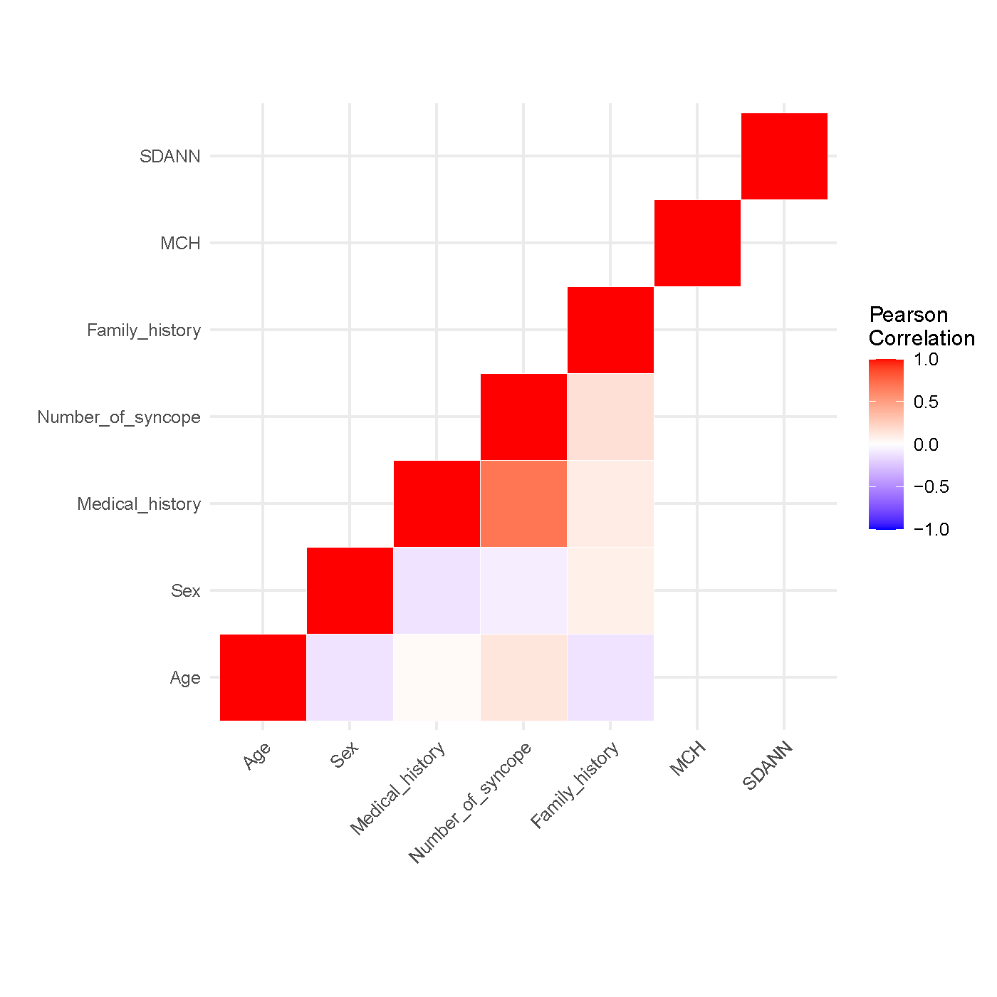


**Figure 1S.** Heat Map Plot of the correlation


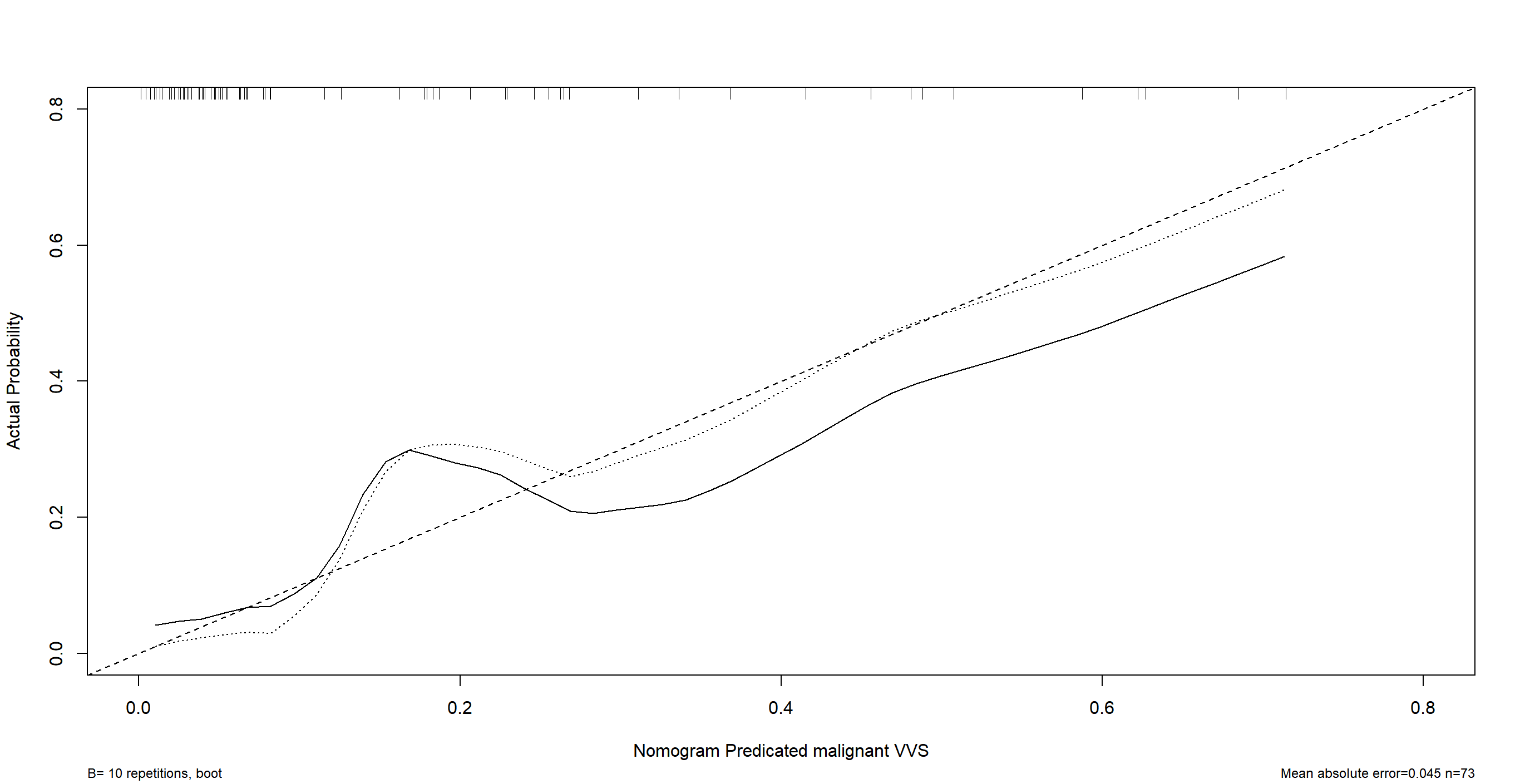


**Figure 2S.** The calibration curve for predicting the risk of malignant vasovagal syncope in the nomogram model.
